# Control of allergic rhinitis in four latin american countries: Rinola study

**DOI:** 10.3389/falgy.2022.980515

**Published:** 2022-08-24

**Authors:** Diana L Silva, Susana de Barayazarra, Antonio Valero, Elizabeth Garcia, Silvia Uriarte, Augusto Peñaranda, Edgardo Chapman, Maria B Garcia, Jaime Ocampo, Viviana Valencia, Sergio Moreno, Silvana Corelli, Belkis Lopez, Luis F Ramírez, Lucía Cecilia Pérez, Edgardo Jares, Carlos D Serrano

**Affiliations:** ^1^ Unidad de Alergologia, Fundación Valle del Lili, Cali, Colombia; ^2^ Departamento de Alergologia, Hospital San Roque, Córdoba, Argentina; ^3^ Unidad De Alergia, Hospital Clínic, IDIBAPS, Barcelona, España; ^4^ Departamento de Alergologia, Unimeq ORL, Bogotá, Colombia; ^5^ Departamento de Alergologia, Hospital Cayetano Heredia, Lima, Perú; ^6^ Unidad De Alergia, Hospital Universitario General Calixto García, La Habana, Cuba; ^7^ Unidad De Alergia, Fundación LIBRA, Buenos Aires, Argentina

**Keywords:** allergic rhinitis, control, Latin America, validated tools, score

## Abstract

**Background:**

Allergic rhinitis (AR) affects up to 40% of the general population, there are large-scale multicenter studies that have described its characteristics and few studies have focused on studying patients with AR in Latin America (LA).

**Methodology:**

A cross-sectional, descriptive, multicenter study was carried out in four LA countries (Colombia, Argentina, Cuba and Peru). Patients diagnosed with AR between November 2017 and June 2020 were included. Sociodemographic and clinical data, sensitization profile and current treatment were collected in the Electronic Data Collection (BDClinic). Patients also filled out this questionnaires: Rhinitis Control Assessment Test (RCAT), Reflexive Total Nasal Symptom Score (rTNSS), Modified ARIA Criteria for AR Severity (mARIA) and ESPRINT-15. Risk of bias was examined by applying the STROBE checklist.

**Results:**

The study included 412 patients. Median age was 25 years (15–39). Two hundred and twenty four (54.3%) were women. Nasal obstruction was present in 303 (73.5%). Three hundred and thirty four (81%) had a persistent AR. One hundred and twenty one (31.3%) had associated asthma. The most frequently positive skin tests were: *Dermatophagoides pteronyssinus* in 365 (88.6%) and *Dermatophagoides farinae* in 331 (81.3%). Four hundred and eleven patients (99%) reported that AR affected their quality of life. The median score of ESPRINT-15 was 1.87 (0.93–2.93), The mean values of RCAT and rTNSS were 19.01 (±4.59) and 5.4 (±2.97) respectively. Two hundred and fifty (60%) were receiving only oral antihistamines. Physicians decided to start nasal corticosteroids in 296 (71.8%). Only seventy patients (16.9%) were receiving immunotherapy.

**Conclusion:**

These findings confirm that most of patients with AR in LA have a persistent disease with a negative impact on quality of life. Dust mites are the main sensitizers. These findings will allow to know the true impact of AR and can lead to a better disease management.

## Introduction

Although there are large-scale multicenter studies that have described its characteristics, few well conducted studies have described the characteristics of the patients with allergic rhinitis (AR) in Latin America. Neffen et al. (1) described the prevalence and impact of the disease in several Latin American countries. In a total of 1,545 patients, the prevalence of AR was 7%, a surprisingly low percentage compared to the ISAAC study (International Study of Asthma and Allergies in Childhood), which reported a prevalence of 27.9% in children aged 6 to 7 years and 37.6% in adolescents aged 13 to 14 years. Much of this difference is explained by the fact that the ISAAC study used questionnaires answered by patients, while the Latin American study was based on physician diagnoses, which implies variation in diagnostic certainty.

The treatment of AR combines education, allergen avoidance and other preventive measures (e.g., lifestyle), pharmacotherapy, and allergen-specific immunotherapy. Pharmacological measures for the management of AR should be determined by the characteristics of the disease and its severity. According to the ARIA document, pharmacological treatment must be individualized for each patient and mainly includes second-generation oral or topical antihistamines (nasal and/or ocular) and intranasal or systemic corticosteroids (2). Intranasal corticosteroids are the most effective means of managing AR and have been shown to be superior to antihistamines and other drugs (3). Although AR has negative consequences when not treated (poor quality of life and disease control, progression of the disease to asthma, and increased use of health resources), approximately 50% of patients with AR do not adhere to treatment (4).

Finally, assessing the control of AR is fundamental for determining the influence of treatment on symptoms, sleep quality, and activities of daily living, their impact on respiratory function, the degree of treatment response, and the impact on exacerbations and prognosis (5).

The objective of the present study was to describe the control and clinical characteristics, sensitization profile, treatment and quality of life of patients with rhinitis and/or allergic conjunctivitis in four Latin American countries.

## Materials and methods

An epidemiological, observational, prospective, multicenter international study was designed. five centers from Argentina, Colombia, Peru, and Cuba participated, the level of healthcare of the first four centers where tertiary, and the level of Cuba center was secondary.

Patients older than six years with AR and/or allergic conjunctivitis with sensitization to one or more clinically relevant aeroallergens were included consecutively and were interviewed both at the first visit but also in the control visits. Individuals with chronic rhinosinusitis with or without polyps or with occlusive nasal septal deviation were excluded. The results of the skin tests were obtained from the clinical history at the control consultations, or in those of first time consultation, these were underwent at that moment (or few days after if taking antihistamines), and their results were included in the study.

The centers of Colombia used the Inmunotek laboratory aeroallergen battery, whose extracts have the following concentrations: Dermatophagoides pteronyssinus (DPT): 100 HEP (histamine equivalent potency, 1 hep = 1000 UB/ml), Dermatophagoides farinae (DPF): 100HEP, Blomia tropicalis (BT) 150 *μ*g/ml, cat epithelium (CE): 50 HEP, dog epithelium (DE): 200 *μ*g/ml, Periplaneta americana (PA): 1000 *μ*g/ml, Alternaria (A): 3 *μ*g/ml, Aspergillus(AP): 25 *μ*g/ml, weeds (W): 103.5 *μ*g/ml, Penicilium (P): 25 *μ*g/ml, Cladosporium (C): 25 *μ*g/ml, Horse epithelium (HE): 50 *μ*g/ml. In the center of Argentina they used allergens from Allergopharma laboratory, with a concentration of whole extracts for aeroallergen used of 100,000 SU/ml. In Cuba the included center used extracts of DPT, D. siboney and BT produced and standardized at the National Center for Biopreparations from Cuba (BIOCEN), at a dilution of 20,000 BU/ml and 100,000 BU/ml respectively, for fungi from Sam Allergeni laboratories at a concentration of 20,000 BU/ml. Lastly, the center of Peru used extracts from the ALK-Abelló laboratories, at the following concentrations: DP, DF: 30 HEP and the BT extract at 10 HEP.

Sociodemographic and clinical data were collected in a database. The following tools were used: Rhinitis Control Assessment Test (RCAT) (6); Reflective Total Nasal Symptom Score (rTNSS) including rhinorrea, nasal itching, sneezing and nasal congestion; (7), Modified ARIA criteria for AR severity (mARIA) (8); ESPRINT-15 questionnaire (9); and in asthmatic patients, the severity and control of asthma (according to Spanish Guide for the Management of Asthma -GEMA- version 5.1) (10). Variables with a symmetric distribution were expressed as the mean and standard deviation, and those with an asymmetric distribution were expressed as the median and interquartile range. The normality of the variables was evaluated by the Shapiro-Wilk test, considering a *p*-value ≤0.05. The data were processed using the statistical package Stata, version 14. Ethics committee of Fundación Valle Del Lili approved the study and the participation of the external centers. Because most patients already had a clinical history with the data and that performance of skin tests were part of the usual consultation of each center, the ethics committee did not required to sign informed consent, but all authors signed a confidentiality agreement to protect identity and personal data. All participating centers followed the same study design. The study followed the Guidelines of Systematic Reporting of Examination in the STROBE checklist. There are 22 main items, each of which was completed.

## Results

Four hundred twelve patients were included: 191 (46%) from Colombia, 105 (26%) from Argentina, 92 (22%) from Peru, and 24 (6%) from Cuba. The median age was 25 years (15–39). Of the total, 224 (54.3%) were women. Demographic characteristics are described in Table 1. At the visit, 301 patients (73.5%) presented with nasal obstruction, 272 (66%) with sneezing, 247 (60.1%) with nasal itching, 243 (59.2%) with rhinorrea, and 101 (24.5%) with loss of smell. Two hundred seventy-two (66%) reported ocular symptoms consistent with allergic conjunctivitis. Of them, 139 (51.1%) had a persistent form.

**Table 1:** Demographic data, clinical characteristics and sensitization profile.

Demographic variables		
	Median	IQR
Age	25	(15–39)
	No. Patients	Percentage
Sex		
Male	188	45,7
Female	224	54,4
Country		
Colombia	191	46,3
Argentina	102	24,7
Peru	92	22,3
Cuba	24	5,8
Comorbidities		
None	268	65
Asthma	129	31,3
Otitis media	9	2,2
Atopic dermatitis	2	0,5
Temporality of rhinitis		
Intermitent	78	18,9
Persistent	334	81,1
Severity of rhinitis		
Mild	91	22,09
Moderate	288	69,9
Severe	33	8,01
Temporality of asthma		
Intermitent	61	47,3
Persistent	68	52,7
Severity of asthma		
Mild	11	8.5
Moderate	54	41.9
Severe	5	3.9
Sensitization profile		
D. Pteronyssinus	365	88,6
D. Farinae	335	81,3
B.Tropicalis	249	60,4
Cat	91	22,1
Dog	88	21,4
Cockroaches	49	11,9
Alternaria	40	9,7
Aspergilus	18	4,4
Pollen of grasses, herbs	25	6,0
Penicilium	18	4,4
Cladosporium	16	3,9
D. Siboney	6	1,5
Horses	5	1,2

IQR, interquartile range.

On skin tests, the most frequently found allergens were *Dermatophagoides pteronyssinus* (DPT) in 365 (88.6%), *Dermatophagoides farinae* (DPF) in 331 (81.3%), *Blomia tropicalis* (BT) in 249 (60.4%), cat epithelium in 91 (22.1%), and dog epithelium in 88 (21.4%), pollens from grasses, herbs and ragweed in 25 patients (6%). (Table 1). Three hundred thirty-four patients (81%) had persistent AR, of which 288 (69.9%) were moderate. A significant association was found between the presence of persistent AR and sensitization to DPT (*p* = 0.044). One hundred twenty-one (31.3%) had associated asthma. A significant association was also found between sensitization to BT and the presence of asthma (*p* = 0.05).

**Figure 1:**
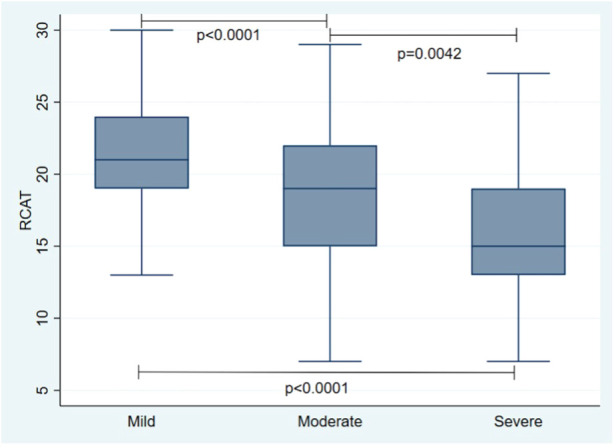
Association between rhinitis control and rhinitis severity.

**Figure 2:**
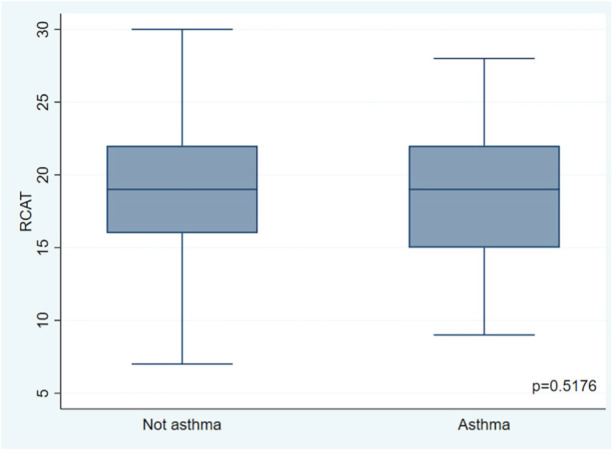
Association between rhinitis control and the presence of asthma.

The mean RCAT score was 19.01 ± 4.59, consistent with partially controlled disease, while the mean rTNSS score was 5.4 ± 2.97. A significant association was found between the presentation of a worse RCAT score and a greater severity of rhinitis. In contrast, no significant association was found between RCAT scores and the presence of asthma **(**Figures 1, 2). Four hundred eleven patients (99%) reported that AR affected their quality of life; 156 (38%) considered the symptoms troublesome and 144 (35%) reported sleep disturbance. The median score on ESPRINT-15 questionnaire was 1.87 (0.93–2.93). A significant association was found between ESPRINT-15 scores greater than three and the presence of sensitization to DPF (*p* = 0.037) and dog epithelium (*p* = 0.026).

Regarding pharmacological treatment, the study found that 250 patients (60.6%) were treated with oral antihistamines, followed by intranasal corticosteroids in 203 (49.3%), and the combination of oral antihistamine and nasal corticosteroid in 150 (36.4%). In a minor percentage, other pharmaceutical interventions were used: nasal decongestants in 38 (9.2%), intranasal antihistamines in 32 (7.8%), montelukast in 28 (6.8%), oral antihistamine plus decongestant in 21 (5%), oral corticosteroids in 9 (2.2%), and the combination of nasal antihistamine plus corticosteroid in 8 (1.9%) patients.

Treating physicians considered that AR was not adequately managed in 245 patients (59.4%) and decided to change the treatment in 217 (52.6%). Only 70 (16.9%) were receiving specific immunotherapy (SIT); 51 of them (72.5%) with house dust mites (HDM) extracts and 65 (92.8%) by subcutaneous way. Physicians considered that 359 patients (87.1%) could be candidates for immunotherapy.

## Discussion

The prevalence of allergic rhinitis has increased in recent years, reaching epidemic proportions in both developed and developing countries. This highlights the importance of knowing and identifying the different clinical and epidemiological characteristics of patients with AR in different geographical areas. In Latin America, there are few studies of the clinical profile of the disease involving several countries (1).

In the present study, there was a clear predominance of AR among females (54.3% of cases), which is similar to the finding of the systematic review by Pinart et al. (11), which aimed to determine the influence of sex on the prevalence of AR. The authors concluded that the change in the prevalence of rhinitis from childhood to adulthood with respect to gender (male and female predominance respectively) could be related to anatomical, physiological, immunological, and hormonal differences during puberty.

Regarding AR symptoms, in the present study, 73.5% of patients reported nasal obstruction, 66% reported sneezing, 60.1% reported nasal pruritus, 59.2% reported nasal discharge, and 24.5% reported loss of smell, findings that were consistent with those reported in other studies (12). Additionally, nasal obstruction was the most troublesome symptom. Shedden et al. (13) conducted a survey of patients with AR and found that 85% of the 2355 included individuals considered nasal obstruction the most troublesome symptom, and almost half of the respondents rated it as severe, with a clear impact on their work performance. Nasal obstruction is considered the most troublesome symptom of AR in different studies and has a significant impact on quality of life due to reduced participation in outdoor sports or social events and the negative impact on emotions, work and school productivity, and sleep. It can also lead to other conditions, such as pain, throat dryness and/or irritation, facial pain or pressure, oral breathing, Eustachian tube dysfunction, and hearing, smell, and/or taste problems. AR-related chronic nasal obstruction has been associated with an increased risk of developing other respiratory tract diseases, such as rhinosinusitis and otitis media (14). However, many people with AR and nasal obstruction do not receive an accurate diagnosis or adequate pharmacological treatment. Considering the effects on the patients lives, it is imperative for the medical community to recognize the impact of AR and establish educational and diagnostic strategies for this disease.

The present study evaluated the impact of both nasal and non-nasal symptoms in patients with AR. Ocular symptoms, particularly ocular pruritus, were the most frequent non-nasal symptom; they were present in 261 patients (63% of the total and 91% of those with ocular symptoms). The vast majority of the patients considered ocular signs and symptoms troublesome and reported that they had a significant impact on their quality of life. Previous studies have reported that the presence of ocular symptoms in patients with AR leads to a loss of productivity, decreases quality of life, and increases the burden on resource use, which highlights the importance of optimal ocular treatment (15, 16).

Regarding the ARIA classification of frequency and severity, most patients (81%) were classified with persistent AR and moderate severity (69.9%), a finding similar to that observed by Neffen et al. (1) and other studies.

In the present study, it was found that HDMs were the main sensitizing allergens, which is consistent with research conducted in Latin America (17–19), Europe (20, 21), and Asia (22, 23). Of the three mites tested, 365 patients (88.6%) were positive for *Der p*, followed by *Der f* in 331 (81.3%) and *Blo t* in 249 (60.4%).

Knowledge of the most relevant aeroallergens in Latin American patients with AR can help to make this growing health problem more visible in the region and can aid in the implementation of preventive measures, environmental controls, and specific immunotherapy for the control of the disease. Additionally, it has been described that the presentation, simultaneity and severity of AR and asthma may be influenced by sensitization to aeroallergens; similarly, the characteristics of AR may also determine the presence and/or pattern of asthma (20, 24).

AR frequently coexists with asthma (25). This is evidenced by the data found in this study, in which 121 patients (31.3%) with AR also presented with asthma. Of these, 68 (52.7%) were classified as persistent asthmatics. These findings support the importance of taking a complete medical history and performing a physical examination, with an emphasis on other potentially affected organs, in patients with AR. Additionally, a significant association was found between the presence of persistent AR and sensitization to *Der* p (*p* = 0.044) and between the presence of persistent asthma and sensitization to *Blo t* (*p* = 0.027). These data suggest that the characteristics of AR may influence the development and persistence of asthma and that allergies to HDM are associated with more lasting and/or severe disease patterns. This supports the concept that respiratory allergic disease is a systemic disease and that AR and asthma are manifestations of the same disease (26), and it suggests that AR severity and sensitization to HDM may be markers of progressive involvement of the lower respiratory tract.

It is well established that AR has a significant impact on quality of life. Individuals with AR not only typically complain about how troublesome AR symptoms are but also manifest a major decrease in emotional well-being and social functioning. A total of 411 patients (99%) reported some impact on their daily life, which is consistent with the reports of other studies (27). The majority of changes in the quality of life of patients with AR are associated with sleep disorders. In this study, 144 (35%) patients had impaired sleep, which has a profound effect on mental health and work and academic performance. In a systematic review with meta-analysis, Liu et al. (28) found that AR was associated with difficulty waking up, daytime sleepiness, morning headache, and the use of sleep medications, which resulted in poor daytime performance. Similar results were found in a study by Stuck et al. (29). The data from the present study reveal a significant association between AR and sleep characteristics and their negative consequences for daytime activity. Additionally, 109 patients (26.5%) presented impaired school or work performance due to AR symptoms; this finding is consistent with the systematic review by Vandenplas et al. (30), which analyzed studies that included the Work Productivity and Activity Impairment (WPAI) questionnaire, used to measure the reduction in productivity associated with specific medical conditions. Those researchers found that among patients with AR, 3.6% missed work time (absenteeism), and 35.9% had impairments in at-work performance (presenteeism). The economic evaluations indicated that the indirect costs associated with productivity loss were the main contributors to the total costs of AR and were a consequence of presenteeism in the majority of cases. The severity of AR symptoms was the factor most frequently related to a greater impact of AR on work productivity.

Considering the large impact of AR on patient quality of life, the present study administered the ESPRINT-15 questionnaire, a validated tool for evaluating health-related quality of life in patients with AR that yields dimension scores ranging from 0 (low impact on quality of life) to 6 (high impact on quality of life). A median score of 1.87 (0.93–2.93) was found, which represents a mild impact on quality of life according to the Spanish validation of this instrument and its reference values (9, 31).

Intranasal corticosteroids (ICs) are the pharmacological management of choice for persistent forms of AR; however, many patients self-medicate, and others are prescribed different drugs with lower evidence and efficacy (32, 33). This study investigated the treatment that patients had been receiving and found that 250 of them (60%) received only oral antihistamines, and 203 (49.2%) received ICs. These data suggest that AR is underestimated by patients themselves and that physicians often do not indicate the treatment of choice. The specialists considered that 245 patients (59.4%) were not adequately managed, and all of them underwent changes in treatment, including the initiation of ICs in 296 (71,8%).

Allergen-specific immunotherapy (SIT) is indicated for patients with moderate to severe AR, either intermittent or persistent, who do not improve despite optimal pharmacological treatment and environmental control measures. It can also be considered in less severe cases in which the patient desires long-term improvement or a potential effect on the progression of their disease (for example, to prevent the development of asthma). Therefore, SIT should be used when available and indicated (41). In this study, only 70 of the patients (16.9%) received SIT. The specialists considered that 359 (87.1%) warranted the initiation of SIT, most often for HDM. The results of several international studies suggest that SIT is a clinically effective and safe treatment that can substantially reduce the cost of pharmacological treatment in allergic patients (34, 35). However, the low percentage of patients with moderate to severe persistent AR who had been receiving SIT suggests that in addition to not providing adequate pharmacological control of AR, the Latin American medical community is unaware of the existence and/or scientific evidence of SIT.

Lastly, it is very useful to evaluate the control of AR since most AR patients tend to underestimate the importance of treatment and the need to control their disease; in addition, they usually seek medical treatment only during exacerbation phases, hoping to obtain quick and transitory relief. Therefore, the use of disease control questionnaires is particularly attractive. Thus, to establish changes in treatment strategies or determine the need for referral to an allergist, the RCAT questionnaire, a brief and practical tool, was developed (36). In the present study, the mean RCAT score was 19.01 ± 4.59, which corresponds to partially controlled disease according to various studies and the recent validation in Spanish (RCATe) (6). Both the RCATe and the psychometric validation showed good reliability, validity and responsiveness, which suggests that these tools are effective for the evaluation of Spanish-speaking patients. In 2018, Zhu et al. (37) showed that adjusting the pharmacological treatment according to the RCAT score resulted in a reduction of costs without compromising control compared to not administering the questionnaire and not changing the treatment. The researchers concluded that the RCAT is a useful tool for guiding efficient step-down pharmacotherapy in patients with AR. That is, the evidence suggests that in addition to evaluating disease control, this questionnaire can be used to establish the course of the disease and reducing the economic cost of AR.

We consider that the main limitations of this study include its retrospective design because we obtain information directly from patients or their medical records; this can lead to information bias. Also this study have the bias associated with the level of healthcare in which the patients were recruited, because most of the patients with mild to moderate disease are treated on the primary care level or are self-treated or not treated at all. Another limitation to consider is not having included other Latin American countries in order to obtain more extensive information. However, it should be highlighted that countries from extreme latitudes of the continent were included, which can give a reflection of the characteristics of the population studied according to the geographic area that had been studied. It would be important to apply the eHealth tools offered by MASK ARIA in this study and in the next studies of RA in LA.

## Conclusion

The findings of this study show that the majority of patients with AR in this four countries of Latin America have persistent disease that impacts their quality of life. House dust mites are the main aeroallergens involved. Most patients do not receive adequate treatment, which suggests that there is still little knowledge about the disease. The data on prevalence, severity, control, sensitization profile, the impact on quality of life, and the treatment profile described here allow the true impact of AR to be recognized and can increase awareness about the optimal approach, thus improving the comprehensive management of the disease.

## Data Availability

The raw data supporting the conclusions of this article will be made available by the authors, without undue reservation.
